# Spontaneous Splenic Rupture in a Diabetic Patient With Klebsiella pneumoniae-Associated Splenic Abscess: A Case Report

**DOI:** 10.7759/cureus.104043

**Published:** 2026-02-22

**Authors:** Nabaa Mohammed S Mahmood, Roda Rashid Mohamed Bin S Alshamsi, Fatema A Albafta, Hind Al Awadhi, Husni Shalak

**Affiliations:** 1 General Practice, Emirates Health Services, Sharjah, ARE; 2 General Practice, Mohammed Bin Rashid University of Medicine and Health Sciences, Dubai Health, Dubai, ARE; 3 General Surgery, University of Ottawa, Ottawa, CAN; 4 General Surgery, McMaster University, Hamilton, CAN; 5 General Surgery, Dubai Hospital, Dubai Health, Dubai, ARE

**Keywords:** diabetes mellitus, klebsiella pneumoniae, sepsis, splenectomy, splenic abscess, spontaneous splenic rupture

## Abstract

Atraumatic splenic rupture (SSR) is rare and usually occurs secondary to trauma. However, infection, particularly with *Klebsiella pneumoniae* in immunocompromised patients such as those with diabetes mellitus, can lead to splenic abscess, sepsis, and rupture. We report the case of a 52-year-old male with diabetes mellitus and ischemic heart disease who developed sepsis from a left diabetic foot infection. His hospital course was complicated by a splenic abscess caused by *K. pneumoniae*, resulting in SSR that necessitated urgent splenectomy. The patient also underwent multiple surgical interventions, including below-knee amputation, and was discharged in stable condition. This case highlights the importance of early recognition and prompt management of splenic complications in high-risk patients, which are critical for survival.

## Introduction

Sepsis is a life-threatening condition characterized by a dysregulated host response to infection, leading to organ dysfunction [1]. Although trauma is the most common cause of splenic rupture, sepsis-related splenic complications are uncommon. Common sources of sepsis include diabetic foot infections, pneumonia, and other localized infections, which may progress to multisystem organ failure or dysfunction of individual organs such as the spleen, liver, or kidneys. In certain cases, the spleen may become susceptible to rupture even in the absence of trauma.

Splenic rupture can also occur due to nontraumatic causes, with approximately 7% of these cases being idiopathic and the remaining 93% attributed to pathological causes. Nontraumatic splenic rupture carries a reported mortality rate of 12% [2].

The diagnosis of atraumatic splenic rupture is often guided by established criteria, such as those proposed by Orloff and Peskin [3], which include the absence of trauma, absence of disease-causing splenic fragility, a normal spleen on gross or histologic examination except for rupture-related changes, and no evidence of perisplenic adhesions [4].

## Case presentation

A 52-year-old male with a history of type 2 diabetes mellitus, ischemic heart disease, and compensated heart failure presented with a six-day history of subjective high-grade fever and chills, accompanied by generalized dizziness and localized pain and swelling of the left foot. He also reported chest pain radiating to the left shoulder, along with a productive cough.

One month prior, he had undergone a left small toe amputation and wound debridement for a diabetic foot infection. His hospitalization was complicated by an episode of acute coronary syndrome, which was successfully managed with percutaneous coronary intervention. He was subsequently scheduled for elective coronary artery bypass grafting.

On physical examination in the emergency department, the patient was hemodynamically stable with warm, dry skin. Vital signs on arrival were as follows: blood pressure 115/80 mmHg, pulse 56 beats per minute, respiratory rate 18 breaths per minute, temperature 37°C, and oxygen saturation 97% on room air. At presentation, the patient did not meet the Systemic Inflammatory Response Syndrome criteria, despite laboratory evidence of systemic infection and organ dysfunction. General surgery was consulted for further evaluation of his lower limb wounds.

Examination of the lower limbs revealed no visible wounds on the right side; the dorsalis pedis pulse was not palpable but audible on Doppler assessment. The left lower limb exhibited a large plantar wound extending dorsally, with diffuse slough, dried necrotic tissue on the plantar surface, and purulent discharge. The dorsalis pedis pulse was absent on palpation but audible on Doppler. Vascular assessment demonstrated biphasic signals in the posterior tibial artery and triphasic signals in the popliteal artery.

Laboratory investigations on admission showed marked inflammatory response, anemia, acute kidney injury, and hyperlactatemia, consistent with sepsis and multiorgan dysfunction (Table 1).

**Table 1:** Laboratory investigations on admission

Parameter	Patient value	Reference range
White blood cell count	43.6 × 10³/µL	4.0-11.0 × 10³/µL
C-reactive protein	245 mg/L	<5 mg/L
Procalcitonin	274.80 ng/mL	<0.05 ng/mL
Hemoglobin	9.4 g/dL	13.0-17.0 g/dL
Hematocrit	30.30%	40-50%
Platelet count	378 × 10³/µL	150-400 × 10³/µL
Troponin	297 ng/L	<14 ng/L
Creatinine	1.74 mg/dL	0.7-1.3 mg/dL
Estimated glomerular filtration rate	46.6 mL/min/1.73 m²	≥90 mL/min/1.73 m²
Lactic acid	4 mmol/L	0.5-2.2 mmol/L
Random blood glucose	194 mg/dL	70-140 mg/dL

**Figure 1:**
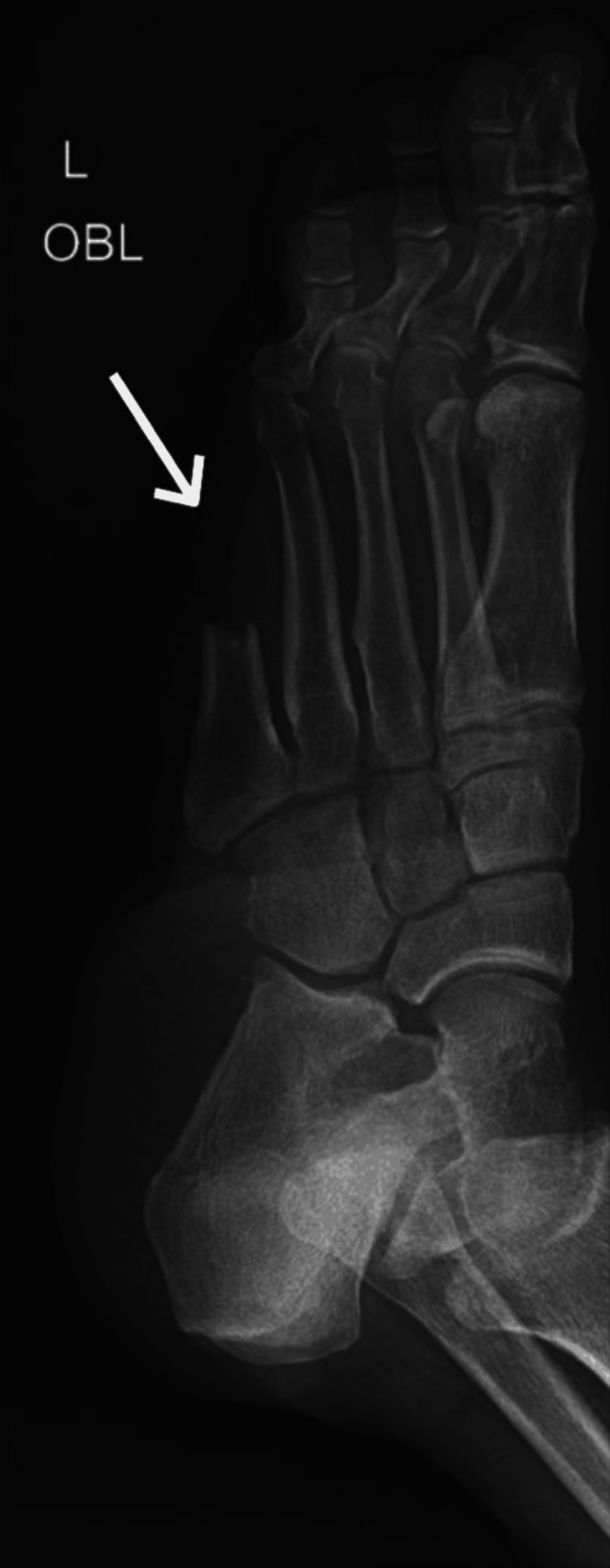
Oblique radiograph of the left foot demonstrating prior surgical changes with non-visualization of the fifth toe phalanges and truncation of the distal fifth metatarsal

An X-ray of the left foot was performed (Figure 1), demonstrating non-visualization of the phalanges of the fifth toe and significant portions of the fifth metatarsal, likely related to prior surgical intervention. No acute fracture or significant bony destruction was identified in the remaining visualized bones. Soft tissue irregularity was noted along the lateral and plantar aspects of the foot. Additionally, atherosclerotic calcifications were observed within the vascular walls.

Based on clinical and laboratory findings, the patient was diagnosed with sepsis secondary to a left diabetic foot infection and was admitted under the general surgery team. Given his severe sepsis, recent healthcare exposure, and high risk for multidrug-resistant Gram-negative organisms, empiric broad-spectrum antimicrobial therapy with ceftazidime-avibactam was initiated on admission, with plans to tailor treatment according to subsequent culture and sensitivity results.

**Figure 2:**
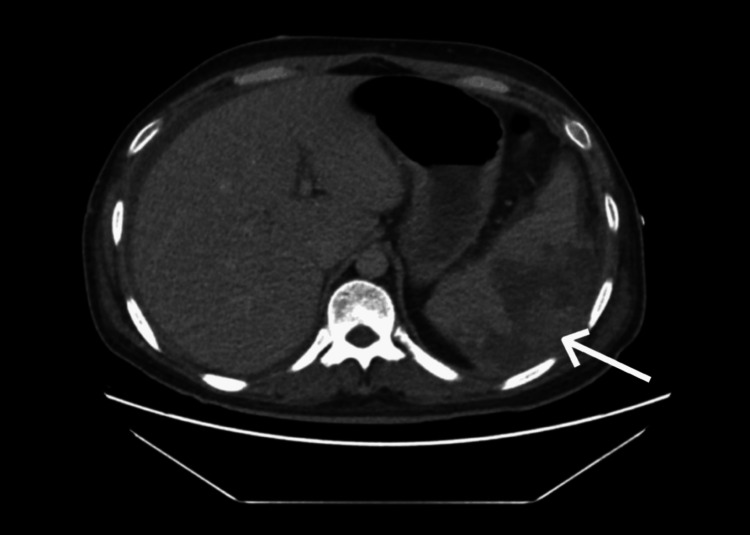
Contrast-enhanced CT scan, axial view of the abdomen, showing splenic abscess rupture

On day 2 of admission, the patient developed acute left upper quadrant abdominal pain and experienced worsening hemodynamics. Clinical examination revealed abdominal rigidity and tenderness in the left upper quadrant. A contrast-enhanced CT scan of the abdomen demonstrated splenomegaly with an ill-defined, heterogeneous, non-enhancing area in the spleen (Figure 2), raising suspicion for a ruptured splenic abscess or hematoma. Additionally, a heterogeneous collection was noted in the spleen, along with free fluid in the perisplenic, perihepatic, and pelvic regions, suggesting a ruptured spleen or hematoma, as well as a left pleural effusion.

Given the presence of intra-abdominal hemorrhage secondary to splenic rupture in the context of ongoing sepsis, his hemodynamic instability was likely multifactorial, reflecting mixed shock physiology with hemorrhagic shock superimposed on septic shock. He was initially transferred to the ICU and started on inotropic support, followed by an emergency laparotomy and splenectomy. Abdominal fluid collected intraoperatively tested positive for *Klebsiella pneumoniae*.

Intraoperative findings included a large volume of turbid intra-abdominal fluid mixed with blood and clots in the subhepatic, suprahepatic, and perisplenic spaces. A ruptured splenic abscess and a 4 cm rupture in the superior pole of the spleen were observed, with surrounding necrotic tissue extending toward the diaphragmatic and gastrosplenic omentum.

Post-splenectomy, the patient was intubated for six days and subsequently underwent a left foot Chopart amputation due to progressive necrosis. Culture from the foot wound grew vancomycin-resistant *Enterococcus faecium*, prompting the addition of cefepime and tigecycline to his antibiotic regimen. Nineteen days after the Chopart amputation, he underwent a below-knee amputation due to exposed tendons, thrombosed vessels, and inadequate soft tissue coverage.

Postoperatively, the patient received vaccinations for pneumococcus and meningococcus and was advised to receive the *Haemophilus influenzae *vaccine, which was not available at our facility. He was transferred to the ward, completed a full course of antibiotics, and was subsequently discharged in stable condition with outpatient follow-up.

## Discussion

Nontraumatic splenic rupture is a rare but potentially fatal event, most frequently associated with hematologic malignancies and systemic infections. This case meets the diagnostic criteria for atraumatic splenic rupture described by Orloff and Peskin [3], including the absence of trauma, absence of underlying splenic disease apart from the abscess, and gross and histologic confirmation of rupture-related changes.

Only a limited number of case reports describe sepsis as an underlying cause of splenic rupture, underscoring both its rarity and high associated mortality. For instance, a case of pneumonia-associated sepsis revealed spontaneous splenic rupture (SSR) incidentally on ultrasound; despite splenectomy, the patient succumbed to respiratory failure [5]. Similarly, *Clostridium perfringens* sepsis during late pregnancy led to catastrophic splenic rupture with massive intra-abdominal hemorrhage, resulting in both maternal and fetal death [6].

Vector-borne infections have also been implicated in splenic rupture, with scrub typhus and malaria, particularly *Plasmodium knowlesi*, reported in endemic regions. These infections have been reported as causes of splenic rupture in elderly patients, often with fatal outcomes [7,8]. More recently, disseminated actinomycosis has been reported to cause splenic rupture with concurrent hepatic abscesses [9].

Our case adds to this growing body of evidence by highlighting splenic rupture secondary to *K. pneumoniae* sepsis originating from a diabetic foot infection. In contrast to previously reported cases with fatal outcomes, our patient survived due to timely diagnosis, emergency splenectomy, and targeted antimicrobial therapy. This emphasizes the importance of early imaging, aggressive infection control, and prompt surgical intervention in improving outcomes for patients with sepsis-related splenic rupture, particularly in high-risk diabetic populations.

Another uncommon but clinically significant splenic pathology is splenic abscess; it is rare in clinical practice, with an estimated incidence of 0.2-0.7% based on autopsy series [10]. However, an increasing incidence has been reported in recent years, likely due to the growing number of immunocompromised patients, including those with HIV, long-term corticosteroid use, chemotherapy exposure, trauma, and malignancy. Additional risk factors include intravenous drug use, diabetes mellitus, and infective endocarditis [11].

In East Asia, particularly in regions with predominantly Southeast Asian populations such as Taiwan and Korea, invasive *K. pneumoniae i*nfections have been increasingly documented over the past three decades [12]. Diabetes mellitus is a well-established risk factor for invasive *K. pneumoniae *infection [13]. Poor glycemic control impairs neutrophil chemotaxis and phagocytosis, reducing pathogen clearance and facilitating the progression of localized infections to bacteremia and deep-seated abscesses, including splenic involvement [14]. In high-risk patients, this immune dysfunction can lead sequentially from uncontrolled blood glucose to severe foot infection, bacteremia, hematogenous seeding of the spleen, abscess formation, and ultimately spontaneous rupture, highlighting the importance of early recognition, glycemic optimization, and timely medical and surgical interventions to prevent life-threatening complications.

Foot infections in patients with diabetes mellitus are commonly referred to as diabetic foot sepsis (DFS). A wide range of microorganisms, either as single pathogens or polymicrobial combinations, have been implicated in DFS. Aerobic gram-positive cocci, particularly* Staphylococcus aureus*, are the most frequently isolated organisms. In chronic or polymicrobial infections, gram-negative aerobic bacilli, including *Pseudomonas aeruginosa* and members of the Enterobacteriaceae family, are commonly identified. Antimicrobial resistance is an increasing concern, with frequent isolation of extended-spectrum β-lactamase-producing Enterobacteriaceae, methicillin-resistant* S. aureus*, resistant *P. aeruginosa *strains, and vancomycin-resistant *E. faecium *in microbiological cultures from patients with DFS. In this case, vancomycin-resistant *E. faecium *was isolated, which may have contributed to the severity of the septic process. Notably, *Enterococcus faecalis *is more commonly isolated and has been reported to exhibit lower resistance rates, estimated at approximately 6.7% [15].

A thorough clinical history, including prior surgeries, hepatic disease, recent infections, anticoagulant or antiplatelet use, and underlying bleeding disorders, is essential when evaluating patients with suspected splenic rupture in the setting of sepsis. Clinically, severely ill patients may present with hypovolemic shock manifested by tachycardia, hypotension, and pallor. Other features include left upper quadrant abdominal tenderness, signs of generalized peritonitis, or referred pain to the left shoulder (Kehr’s sign) [16].

Pathogenic organisms can disseminate hematogenously and replicate within highly vascular organs such as the spleen, heart, and kidneys. The spleen and other secondary lymphoid organs serve as reservoirs for immune cells, including macrophages and B and T lymphocytes, and play a critical role in immune surveillance and blood filtration. This immunologic function renders the spleen particularly vulnerable to infectious injury, potentially leading to organ dysfunction, abscess formation, or, in severe cases, autosplenectomy.

During sepsis, the spleen’s role in antigen-presenting cell and lymphocyte interactions becomes especially important. Impairment of these interactions results in inadequate microbial clearance and increases the risk of abscess formation [17]. Further studies are needed to elucidate the role of splenic lymphocyte and macrophage populations in the pathogenesis of splenic abscesses and the subsequent risk of rupture.

Disseminated infections involving vital organs require immediate intervention to prevent life-threatening complications. Early diagnosis, aided by imaging, and initiation of broad-spectrum antimicrobial therapy are essential in managing splenic abscesses or hematomas. While antibiotics play a critical role in infection control, they are often insufficient as monotherapy to prevent rupture. In selected patients, spleen-preserving approaches such as image-guided percutaneous drainage may be considered to maintain immunologic function [18]. However, in cases complicated by spontaneous rupture, emergency laparotomy is indicated to prevent contamination of sterile body cavities by infected hematoma or abscess contents.

Splenectomy remains the definitive management for ruptured splenic abscess. Post-splenectomy immunization is essential to reduce the risk of overwhelming post-splenectomy infection, as the spleen is crucial for mounting immune responses against encapsulated organisms. Vaccination against *Streptococcus pneumoniae*, *H. influenzae*, and *Neisseria meningitidis *is therefore recommended [19], which explains the immunization regimen administered to this patient following splenectomy.

Beyond splenectomy, attention should also be directed toward the optimal timing of interventions addressing the primary source of infection. Based on this case, earlier below-knee amputation may have potentially reduced the progression of sepsis and lowered the risk of subsequent splenic abscess formation and rupture. However, the patient’s underlying comorbidities, including type 2 diabetes mellitus and ischemic heart disease, likely contributed to disease progression and infectious complications. Therefore, although early surgical source control is critical, the patient’s overall health status and the virulence of the infecting organism also play significant roles in determining clinical outcomes [11].

## Conclusions

This case highlights the potential for severe complications related to sepsis in patients with diabetes mellitus, ranging from invasive *K. pneumoniae* infection to the rare occurrence of SSR. It demonstrates that timely diagnosis, early initiation of broad-spectrum antimicrobial therapy, and prompt surgical intervention are critical in limiting morbidity and mortality. In this patient, disease progression from a localized diabetic foot infection to systemic sepsis and subsequent splenic rupture underscores the aggressive clinical course that may occur when infections are inadequately controlled, particularly in individuals with poor glycemic control.

Furthermore, this case emphasizes the importance of long-term management following splenectomy. Asplenic patients are at increased risk of severe infections caused by encapsulated organisms, including* S. pneumoniae*, *H. influenzae*, and *N. meningitidis*. Preventive strategies should therefore include timely vaccination against these pathogens, consideration of antibiotic prophylaxis in high-risk individuals, and patient education regarding early recognition of infection. Overall, this case reinforces the systemic consequences of what may initially appear to be a localized diabetic foot infection and highlights the need for heightened clinical vigilance and a multidisciplinary approach to improve patient outcomes.
